# Genomic Analysis of *Delftia tsuruhatensis* Strain TR1180 Isolated From A Patient From China With In4-Like Integron-Associated Antimicrobial Resistance

**DOI:** 10.3389/fcimb.2021.663933

**Published:** 2021-06-17

**Authors:** Cong Cheng, Wangxiao Zhou, Xu Dong, Peiyao Zhang, Kexin Zhou, Danying Zhou, Changrui Qian, Xi Lin, Peizhen Li, Kewei Li, Qiyu Bao, Teng Xu, Junwan Lu, Jun Ying

**Affiliations:** ^1^ Vocational and Technical College, Lishui University, Lishui, China; ^2^ Institute of Biomedical Informatics, School of Laboratory Medicine and Life Sciences, Wenzhou Medical University, Wenzhou, China; ^3^ Key Laboratory of Medical Genetics of Zhejiang Province, Key Laboratory of Laboratory Medicine, Ministry of Education, China, School of Laboratory Medicine and Life Sciences, Wenzhou Medical University, Wenzhou, China; ^4^ Institute of Translational Medicine, Baotou Central Hospital, Baotou, China

**Keywords:** *Delftia tsuruhatensis*, antimicrobial susceptibility profile, pan-genome, whole-genome analysis, comparative genomics analysis, In4-like integron

## Abstract

*Delftia tsuruhatensis* has become an emerging pathogen in humans. There is scant information on the genomic characteristics of this microorganism. In this study, we determined the complete genome sequence of a clinical *D. tsuruhatensis* strain, TR1180, isolated from a sputum specimen of a female patient in China in 2019. Phylogenetic and average nucleotide identity analysis demonstrated that TR1180 is a member of *D. tsuruhatensis*. TR1180 exhibited resistance to β-lactam, aminoglycoside, tetracycline and sulphonamide antibiotics, but was susceptible to phenicols, fluoroquinolones and macrolides. Its genome is a single, circular chromosome measuring 6,711,018 bp in size. Whole-genome analysis identified 17 antibiotic resistance-related genes, which match the antimicrobial susceptibility profile of this strain, as well as 24 potential virulence factors and a number of metal resistance genes. Our data showed that *Delftia* possessed an open pan-genome and the genes in the core genome contributed to the pathogenicity and resistance of *Delftia* strains. Comparative genomics analysis of TR1180 with other publicly available genomes of *Delftia* showed diverse genomic features among these strains. *D. tsuruhatensis* TR1180 harbored a unique 38-kb genomic island flanked by a pair of 29-bp direct repeats with the insertion of a novel In4-like integron containing most of the specific antibiotic resistance genes within the genome. This study reports the findings of a fully sequenced genome from clinical *D. tsuruhatensis*, which provide researchers and clinicians with valuable insights into this uncommon species.

## Introduction

*Delftia* is a genus of opportunistic, Gram-negative, aerobic, motile, non-spore-forming and nonfermenting bacilli belonging to the family *Comamonadaceae* (Wen et al., 1999). Members of the genus were initially classified into the genus *Comamonas* until 1999, when Wen et al., reevaluated their taxonomy and proposed a new genus named *Delftia* based on phylogenetic and phenotypic evidence (Wen et al., 1999). This genus is grouped into six species: *D. acidovorans* (Wen et al., 1999), *D. tsuruhatensis* (Shigematsu et al., 2003), *D. lacustris* (Jørgensen et al., 2009), *D. litopenaei* (Chen et al., 2012), *D. deserti* (Li et al., 2015) and *D. rhizosphaerae* (Carro et al., 2017), which reside in soil, water, sludge and human microflora (Lipuma et al., 2015). In addition, *D. acidovorans* confers innate resistance to aminoglycosides (Lipuma et al., 2015), which are drugs used as an empirical treatment option for most Gram-negative infections.

*D. tsuruhatensis* was first reported in Japan by Shigematsu et al., in 2003 (Shigematsu et al., 2003). This species was isolated from activated sludge and identified as a terephthalate-assimilating bacterium. Distinguishing *D. tsuruhatensis* from closely related species, such as *D. acidovorans*, using commercial biochemical systems is challenging, thus 16S rRNA gene sequencing is recommended for accurate identification of *D. tsuruhatensis* isolates (Preiswerk et al., 2011). This species is mainly studied for environmental applications, since it can biodegrade organic pollutants, such as phenolic compounds and chlorobenzene (Jimenez et al., 2012; Ye et al., 2019). The species inhibits growth of various plant pathogens and thus is referred as a plant growth-promoting bacterium (Han et al., 2005). Moreover, some *D. tsuruhatensis* isolates have exhibited anti-biofilm activities (Singh et al., 2017) and resistance to heavy metals, such as zinc, lead and cadmium (Dorian et al., 2012; Lin et al., 2016).

Although *D. tsuruhatensis* has been rarely associated with human infections, the microorganism is a possible causative pathogen for life-threatening infections in immunocompromised patients and patients with port-related infections (Preiswerk et al., 2011; Tabak et al., 2013; Ranc et al., 2018). In early 2011, Preiswerk et al., reported a catheter-related infection caused by *D. tsuruhatensis* in a 53-year-old female from Switzerland (Preiswerk et al., 2011). The isolate was resistant to ampicillin, cefuroxime, cefalothin, gentamicin, tobramycin, amikacin and colistin but susceptible to amoxicillin-clavulanate, piperacillin-tazobactam, the third- and fourth-generation cephalosporins, fluoroquinolones and carbapenems. Similar resistance phenotypes were observed in a clinical *D. tsuruhatensis* strain collected from a 53-year-old woman with breast cancer (Tabak et al., 2013). However, a recent study by Ranc *et al.* on the antimicrobial susceptibility of *D. tsuruhatensis* isolated from bronchial samplings of a premature infant from France (Ranc et al., 2018), reported that the strain was categorized as resistant to amoxicillin–clavulanate (MIC >256 mg/L), which was suggestive of the potential evolution of drug resistance for *D. tsuruhatensis* strains; moreover, Ranc et al. found 11 healthcare-associated infections caused by *D. tsuruhatensis* strains through case investigations in the university hospitals in Marseille, France, during 2008-2015 and in the literature, which were mainly isolated from blood cultures (5/11 cases) and respiratory specimens (5/11), but also from a urine sample. And patients in whom *D. tsuruhatensis* had been isolated from blood cultures all had an intravascular device (Ranc et al., 2018).

Currently, there are seven genome sequences of *D. tsuruhatensis* available in the NCBI genome database, including a complete genome of *D. tsuruhatensis* CM13 (accession number CP017420) isolated from murine proximal colonic tissue. However, the specific genomic characteristics of *D. tsuruhatensis* CM13 and the drug resistance of these *D. tsuruhatensis* strains have not been reported. In this study, we analyzed the genomic features of the clinical *D. tsuruhatensis* strain TR1180 with resistance to multiple antibiotics to characterize drug/metal resistance, potential virulence factors and other functional annotations of this strain. We also performed extensive comparative genomics analysis of *D. tsuruhatensis* TR1180 with other sequenced *Delftia* strains to elucidate the genome divergence of these microorganisms at the genus level.

## Materials and Methods

### Bacterial Identification

*D. tsuruhatensis* TR1180 wild-type strain was isolated in 2019 from the sputum of a 91-year-old female patient with respiratory failure at the Central Hospital of Lishui City, China. The strain was initially identified using the Vitek-60 microorganism autoanalysis system (BioMerieux Corporate, Craponne, France). Considering the challenges in discriminating closely related species in the genus of *Delftia* by commercial biochemical systems (Preiswerk et al., 2011), subsequent verification was carried out by phylogenetic analysis of *D. tsuruhatensis* TR1180 with other closely related species. A 16S RNA gene-based tree was generated by MEGA X (Kumar et al., 2018) using neighbor-joining method with 1,000 bootstrap replicates and a whole genome-based tree was constructed using the Type Strain Genome Server (TYGS) (Meier-Kolthoff and Göker, 2019). Further, the identity of the strain was verified by calculating the average nucleotide identity (ANI) values using OrthoANI (Lee et al., 2016).

### Antimicrobial Susceptibility Testing

The minimum inhibitory concentrations (MICs) were determined using the standard agar dilution method following the Clinical and Laboratory Standards Institute (CLSI) guidelines. Susceptibilities to each antimicrobial agent were determined according to the interpretive criteria for “other non-*Enterobacteriaceae*” and “*Enterobacteriaceae*” of CLSI (2019) guidelines. The resistance breakpoints for florfenicol (≥32 mg/L) and streptomycin (≥32 mg/L) were defined as reported in a previous study describing antimicrobial resistance (AMR) of commensal *Escherichia coli* (Wasyl et al., 2013) and the US Food and Drug Administration (FDA), respectively. *E. coli* ATCC 25922 was used as the reference strain for quality control.

### Genome Sequencing, Assembly, and Bioinformatic Analyses

Oxford Nanopore and Illumina HiSeq paired-end sequencing (400-bp insert sizes) of the *D. tsuruhatensis* TR1180 genome were performed. The long nanopore sequencing reads were initially assembled by Canu v1.8 (Koren et al., 2017), then the processed Illumina reads (adaptor trimming and quality filtering) were mapped onto the primary assembly to correct the consensus using BWA-0.7.17 and Pilon v1.23 (Li, 2013; Walker et al., 2014). Potential open reading frames (ORFs) were predicted using Prodigal v2.6.3 (Hyatt et al., 2010), and annotated using the Basic Local Alignment Search Tool (BLAST) (https://blast.ncbi.nlm.nih.gov/Blast.cgi) program against UniProt/Swiss-Prot and non-redundant protein databases with an *E*-value cutoff of 1e-5. EggNOG-mapper v1.0.3 (Huerta-Cepas et al., 2017) was used to assign ORFs to Clusters of Orthologous Groups of proteins (COG) categories by searches against the eggNOG database (Huerta-Cepas et al., 2019). Gene Ontology (GO) and functional pathway annotations of genes were performed using InterProScan-5.36-75.0 and KAAS (Moriya et al., 2007), respectively. The AMR genes, virulence factors, two-component system (TCS) genes and metal resistance genes were predicted using the CARD (Jia et al., 2017), VFDB (Liu et al., 2019), P2CS (Ortet et al., 2015) and BacMet (Pal et al., 2014) with BLASTP (minimum 60% identity and 80% coverage), respectively. RNAmmer 1.2 (Lagesen et al., 2007), tRNAscan-SE 2.0 (Lowe and Chan, 2016) and Infernal 1.1.2 (Nawrocki et al., 2009) were used to identify rRNA, tRNA and noncoding RNA (ncRNA) genes, respectively. Detection of insertion sequences was performed using ISfinder (Siguier et al., 2006). Clustered regularly interspaced short palindromic repeat (CRISPR) arrays were detected using the CRISPRFinder (Grissa et al., 2007). The genome-wide comparison of orthologous genes was performed using GET_HOMOLOGUES (Contreras-Moreira and Vinuesa, 2013). Pan-genome analysis of *Delftia* based on the orthologous genes was performed by PanGP v1.0.1 (Zhao et al., 2014). The graphical map of the *D. tsuruhatensis* TR1180 genome, which was subsequently employed as the reference genome in comparative analysis, was constructed by BRIG v0.95 (Alikhan et al., 2011). The completeness of the genome assemblies was assessed using BUSCO v4.0.5 (Seppey et al., 2019). Comparison of nucleotide sequences was performed using BLASTN. Statistical analysis was performed using R software (https://www.r-project.org/). Other bioinformatics scripts were written using Python (https://www.python.org/) and Biopython (Cock et al., 2009).

## Results and Discussion

### Identification and Antibiotic Susceptibilities of *D. tsuruhatensis* TR1180 Strain

**Figure 1:**
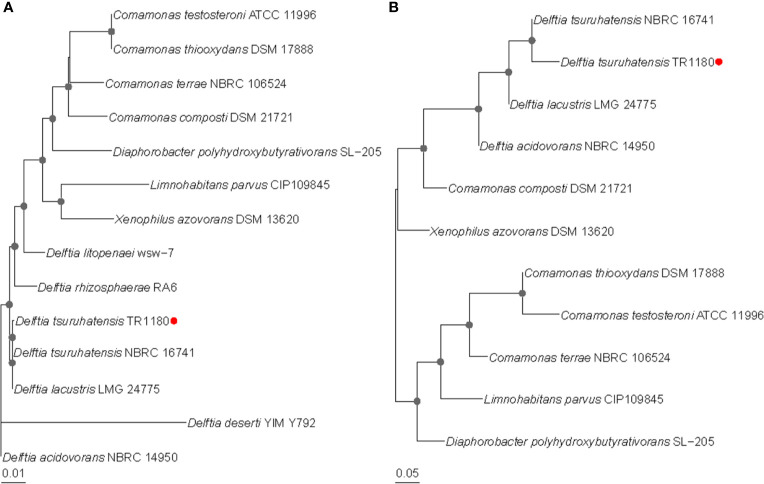
Phylogenetic relationships of *D. tsuruhatensis* TR1180 with other type strains of *Delftia* species and genera within the family *Comamonadaceae*. Phylogenetic trees were based on **(A)** 16S RNA gene sequences and **(B)** whole-genome sequences. Bootstrap values greater than 50% are indicated by gray dots at the nodes. *D. tsuruhatensis* TR1180 is highlighted with a red dot.

A clinical *D. tsuruhatensis* strain designated TR1180 was isolated from the Central Hospital of Lishui City, China. Phylogenetic trees based on the 16S rRNA gene and whole-genome sequences showed that TR1180 belonged to the genus *Delftia* and exhibited the closest evolutionary relationship with *D. tsuruhatensis* NBRC 16741 (NZ_BCTO00000000) (**Figure 1**). Further, a search against the NCBI nucleotide database revealed that TR1180 shared the highest sequence similarity (at 99% identity and 91% coverage) with *D. tsuruhatensis* CM13. The ANI values between TR1180 and the above two *D. tsuruhatensis* strains were 98.09% (NBRC 16741) and 98.01% (CM13), respectively, which exceeded the threshold of 95-96% for species circumscription (Richter and Rosselló-Móra, 2009). Therefore, the study strain was grouped into the species *D. tsuruhatensis*.

Antimicrobial susceptibility testing showed that *D. tsuruhatensis* TR1180 exhibited a multidrug-resistant (MDR) phenotype, including resistance to β-lactams (ampicillin and cefazolin), all tested aminoglycosides, tetracycline (tetracycline) and sulphonamide (trimethoprim/sulfamethoxazole) (**Table 1**). As mentioned earlier, to our knowledge, only three studies have reported the AMR profiles of clinical *D. tsuruhatensis* isolates (Preiswerk et al., 2011; Tabak et al., 2013; Ranc et al., 2018). Our work represented the first study that determined the MICs of phenicol, tetracycline, sulphonamide and macrolide antibiotics for clinical *D. tsuruhatensis*.

**Table 1:** Antimicrobial susceptibility of *D. tsuruhatensis* strain TR1180.

Antibiotic class	Antibiotics tested	MIC (mg/L)	Interpretation
β-lactam	Ampicillin	128	R
	Cefazolin	128	R
	Cefoxitin	4	S
	Ceftriaxone	8	S
	Ceftazidime	0.25	S
	Cefepime	4	S
	Aztreonam	0.5	S
	Imipenem	0.25	S
Aminoglycoside	Streptomycin	>1,024	R
	Gentamicin	>1,024	R
	Tobramycin	>256	R
	Amikacin	256	R
	Netilmicin	>256	R
Phenicol	Chloramphenicol	8	S
	Florfenicol	4	S
Fluoroquinolone	Ciprofloxacin	0.03	S
	Levofloxacin	2	S
Tetracycline	Tetracycline	16	R
Sulphonamide	Trimethoprim/sulfamethoxazole	>1,024	R
Macrolide	Azithromycin	16	S

### General Features of the *D. tsuruhatensis* TR1180 Genome

**Figure 2:**
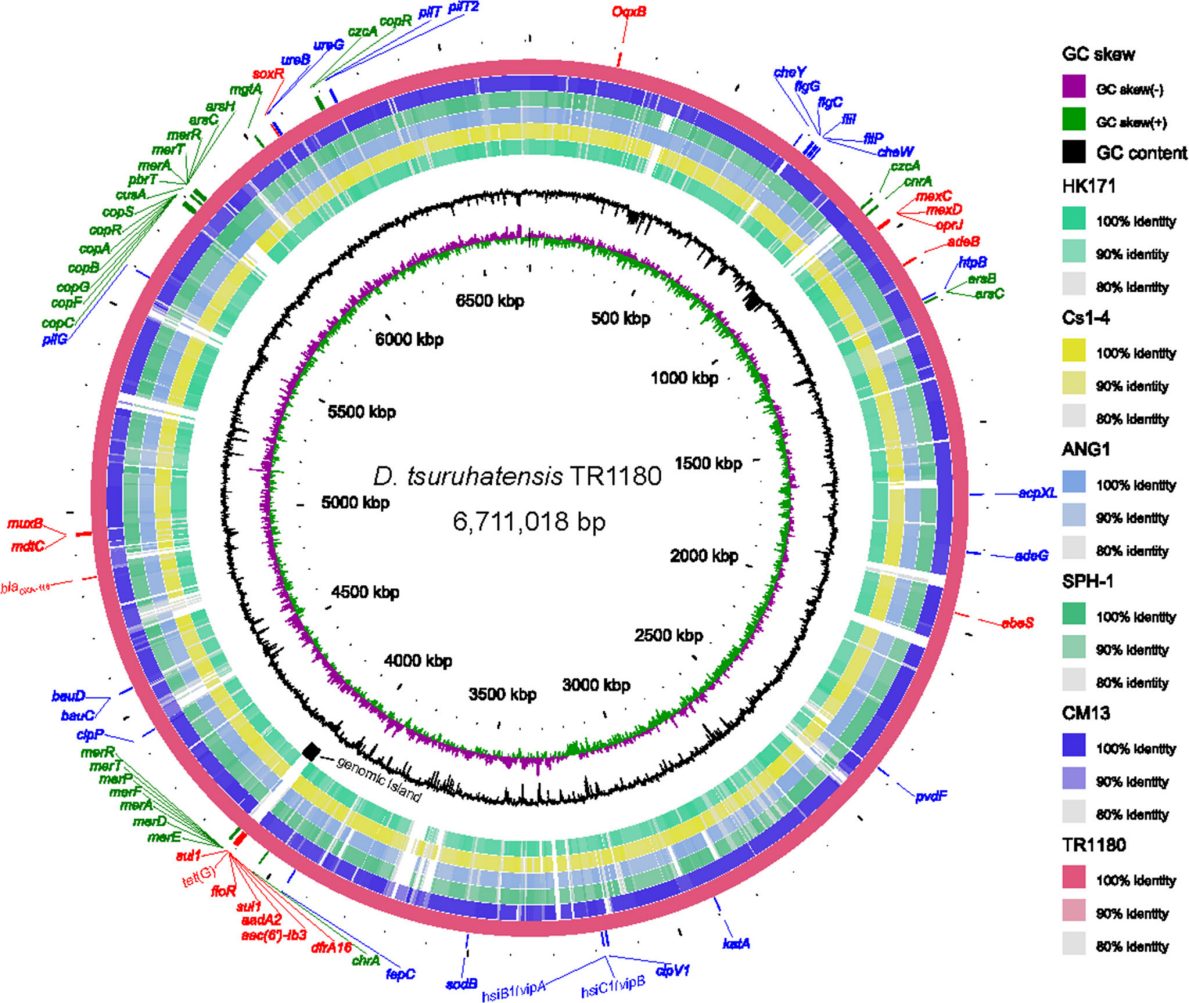
Circular representation of the *D. tsuruhatensis* TR1180 genome and comparative genomics analysis with other *Delftia* strains generated by BRIG. Counting from the outside toward the center: circle 1 shows AMR genes (colored in red), virulence genes (colored in blue) and metal resistance genes (colored in green) encoded on the TR1180 genome; circle 2 indicates the complete genome of TR1180 (as the reference genome); circles 3-7 refer to regions of *D. tsuruhatensis* CM13 (CP017420), *D. acidovorans* SPH-1 (CP000884), *D. acidovorans* ANG1 (CP019171), *D.* sp. Cs1-4 (CP002735) and *D.* sp. HK171 (CP018101) which have high sequence similarities (>80%) with *D. tsuruhatensis* TR1180, where empty regions indicate parts without similar hits between them; circle 8 displays the specific genomic island in TR1180; circles 9 and 10 represent GC content and GC skew of TR1180, respectively.

*D. tsuruhatensis* TR1180 genome was assembled into one circular chromosome of approximately 6.7 Mb in size with an average GC content of 66.52%, and there was no evidence of plasmids (**Table 2** and **Figure 2**). The *D. tsuruhatensis* TR1180 genome harbored 6,009 protein-coding genes, including 17 antimicrobial genes [*bla*_OXA-118_, *oqxB*, *dfrA16*, *aac(6’)-Ib3*, *aadA2*, 2*sul1*, *floR*, *tet*(G) and other multidrug resistance genes], 24 putative virulence genes (mainly related to bacterial motility and adherence, iron acquisition and metabolism, and secretion system) (**Table 3**). In addition, the strain contained a series of predicted metal resistance genes (mercury, copper, arsenate, lead, chromate and other resistance) clustered or scattered on the chromosome (**Table 3**). The presence of drug-resistance genes in the TR1180 genome is in agreement with the antimicrobial susceptibility profile of the strain, except for its susceptibility to phenicols (MIC levels for chloramphenicol and florfenicol were 8 and 4 mg/L, respectively, **Table 1**). The susceptibility to phenicols implies that the *floR* gene in TR1180 may not be functional. Notably, more than half of the virulence genes (62.5%, 15/24) detected in the TR1180 genome are members of offensive virulence factors (**Table 3**). These genes include *acpXL* gene involved in lipopolysaccharide biosynthesis, which may have harmful effects on immunocompromised hosts. Analysis of TR1180 genome identified 15 rRNAs, 87 tRNAs and 14 ncRNAs (**Table 2**). Functional analysis using antiSMASH (Blin et al., 2019) identified a large number of gene loci responsible for the synthesis of the polyketide, nonribosomal peptide, terpene, bacteriocin and resorcinol. Resorcinol has been reported to participate in nematocidal and antimicrobial activity (Sultana et al., 2010; Calderón et al., 2014). The results of COG and GO annotation for TR1180 genome were shown in the **Supplementary Figures S1A, B**, respectively.

**Table 2:** General features of *D. tsuruhatensis* TR1180 genome.

Attribute	Number
Genome size (bp)	6,711,018
GC content (%)	66.52
ORFs	6,009
Known proteins	5,153
Hypothetical proteins	856
Protein coding (%)	89.11
Average ORF length (bp)	995
Average protein length (aa)	330
Genes (RNA)	116
Ribosomal RNAs (rRNAs)	15
Transfer RNAs (tRNAs)	87
Noncoding RNAs (ncRNAs)	14
CRISPR repeats	3

**Table 3:** AMR, virulence and metal resistance genes identified in the *D. tsuruhatensis* TR1180 genome.

Category	Classification of resistance/virulence genes	Related genes
Antimicrobial gene	β-Lactam	*bla*_OXA-118_
	Aminoglycoside	*aac(6’)-Ib3*, *aadA2*
	Phenicol	*floR*
	Fluoroquinolone	*OqxB*
	Tetracycline	*tet*(G)
	Sulphonamide	2*sul1*
	Trimethoprim	*dfrA16*
	Multidrug	*mexC*, *mexD*, *oprJ*, *adeB*, *abeS*, *soxR*, *mdtC*, *muxB*
Virulence gene	Flagella (offensive virulence factors)	*cheY*, *cheW*, *flgC*, *flgG*, *fliI*, *fliP*
	Type IV pili (offensive virulence factors)	*pilG*, *pilT*, *pilT2*
	Type VI secretion system (offensive virulence factors)	*clpV1*, *hsiC1*/*vipB*, *hsiB1*/*vipA*
	AdeFGH efflux pump/transport autoinducer (offensive virulence factors)	*adeG*
	LPS (offensive virulence factors)	*acpXL*
	Hsp60 (offensive virulence factors)	*htpB*
	Acid resistance (defensive virulence factors)	*ureB*, *ureG*
	Iron uptake system (nonspecific virulence factor)	*bauC*, *bauD*, *fepC*, *pvdF*
	Stress protein (nonspecific virulence factor))	*sodB*, *clpP*, *katA*
Metal resistance gene	Mercury	*merE*, *merD*, 2*merA*, *merF*, *merP*, 2*merT*, 2*merR*
	Copper	*copA*, *copB*, *copC*, *copF*, *copG*, 2*copR*, *copS*, *cusA*
	Arsenate	*arsB*, 2*arsC*, *arsH*
	Lead	*pbrT*
	Chromate	*chrA*
	Magnesium	*mgtA*
	Nickel-cobalt	*cnrA*
	Cobalt-zinc-cadmium	2*czcA*

### Pan-Genome Analysis of *Delftia* spp.

To explore the genome divergence and bacterial evolution of the genus *Delftia*, we performed pan-genome analysis of all *Delftia* genomes publicly available in the NCBI genome database (genome completeness ≥95%, **Supplementary Table S1**). The pan-genome possessed 15,316 gene families and had not reached saturation based on its accumulation curve (**Supplementary Figure S2A**). According to the fitted-line model generated by PanGP (Zhao et al., 2014), the *Delftia* pan-genome was open and evolving, indicating species of this genus can colonize different environments and have various ways of exchanging genetic material (Medini et al., 2005). Among these gene families, a total of 2,905 families existed in all thirty-one *Delftia* genomes and hence represented the core genome (**Figures S2B**). The percentages of core genes of each *Delftia* strain ranged from 46.57% to 58.05% (**Supplementary Figure S3**), implying that the core genes comprised a large proportion of the total genes in each genome and hence played a key role in the stability and evolution of the genus *Delftia*. The unique genes (strain-specific genes) are acquired through horizontal gene transfer (HGT) among species to increase genetic diversity and accelerate genome innovation and evolution (Jain et al., 2003). In our study, the number of unique genes across different genomes exhibited a wide distribution, varying from 3 (0.05%) in *D. acidovorans* ANG1 to 1,025 (16.83%) in *D. acidovorans* B15 (**Supplementary Figure S3**). This finding suggests the diverse genetic evolution in different *Delftia* strains. Of the 146 strain-specific genes identified in TR1180 genome, 39.04% were annotated as hypothetical proteins. Therefore, further analysis should be performed to understand the specific functions of these hypothetical genes in TR1180.

**Figure 3:**
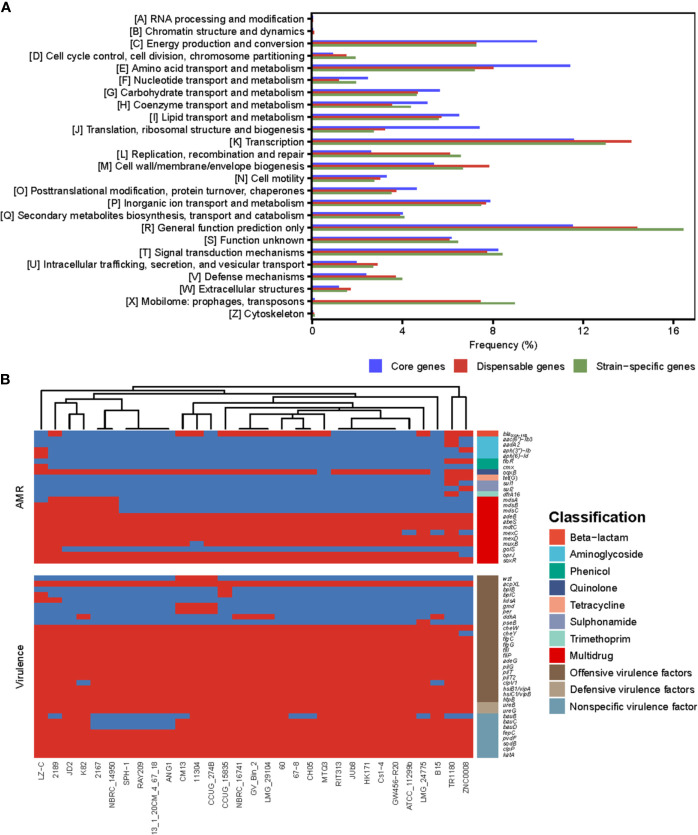
**(A)** The COG function classification comparison of core genes, dispensable genes and unique genes in the genus *Delftia*. **(B)** The resistome and virulome of 31 *Delftia* genomes.

COG annotations were assigned to the core, dispensable and unique genes of *Delftia* strains to better understand the functional differences among them (**Figure 3A**). The result showed that most of the genes in the core genome were involved in housekeeping functions, such as energy production and conversion (9.95%), translation, ribosomal structure and biogenesis (7.42%) as well as transport- and metabolism-related functions (43.02%), and were significantly higher in proportion (all P<0.01; chi-square test) than dispensable (7.27%, 3.21% and 34.68%) and unique genes (7.26%, 2.72% and 35.28%). Compared with core genes, dispensable and unique genes were enriched in functions poorly characterized (14.38% and 16.44%) and functions associated with survival of bacteria in different environments, such as cell envelope biogenesis (7.83% and 6.67%) and mobilome (7.46% and 8.99%). The bacteria cell envelope is a typical example of a complex multilayered structure, which in most bacteria is essential for protecting them from changing and often hostile environment (Silhavy et al., 2010). Substantial mobilome suggests frequent HGT events in *Delftia* genomes, which allow the bacteria to adapt to a new niche or to be more successful in its current niche (Perry and Wright, 2013). These findings support the notion that some dispensable and unique genes in *Delftia* genomes may also be related to niche adaptation.

We also reconstructed the *Delftia* metabolic pathways using the pan-genome and found, although core genes were only significantly enriched in ribosome and oxidative phosphorylation (both FDR<0.01; Fisher′s exact test), several of them were assigned into biofilm formation, beta-lactam resistance and two-component system (TCS) (**Supplementary Table S2**), which were closely associated with pathogenicity and resistance of *Delftia* strains. All *Delftia* genomes shared a similar virulence pattern as shown in **Figure 3B**, and 20 of 33 virulence factors identified in the pan-genome were significantly enriched in the core genome (P<0.01, **Supplementary Table S3**). Interestingly, although most of the AMR genes in these *Delftia* genomes exhibited multidrug resistance (mainly related to antibiotic efflux pumps), some of them conferring resistance to a specific class of antimicrobials were co-located on *D. tsuruhatensis* TR1180, *D.* sp. ZNC0008 and *D. lacustris* LZ-C (**Figure 3B**). This suggested the potential presence of HGT events or mutations among the three *Delftia* genomes. TCS, which is typically composed of a histidine kinase and a cognate response regulator in bacteria, was reported to be involved in the virulence and AMR responses of opportunistic bacterial pathogens (Stephenson and Hoch, 2002). In this study, we identified 157 TCS genes in the pan-genome and found 72 of them were significantly enriched in the core genome (P<0.01, **Supplementary Table S3**). These findings indicated that the core genome of *Delftia* spp. could contribute to pathogenicity and resistance of *Delftia* strains.

### Comparative Genomics Analysis of the Genomic Island in *D. tsuruhatensis* TR1180

**Figure 4:**
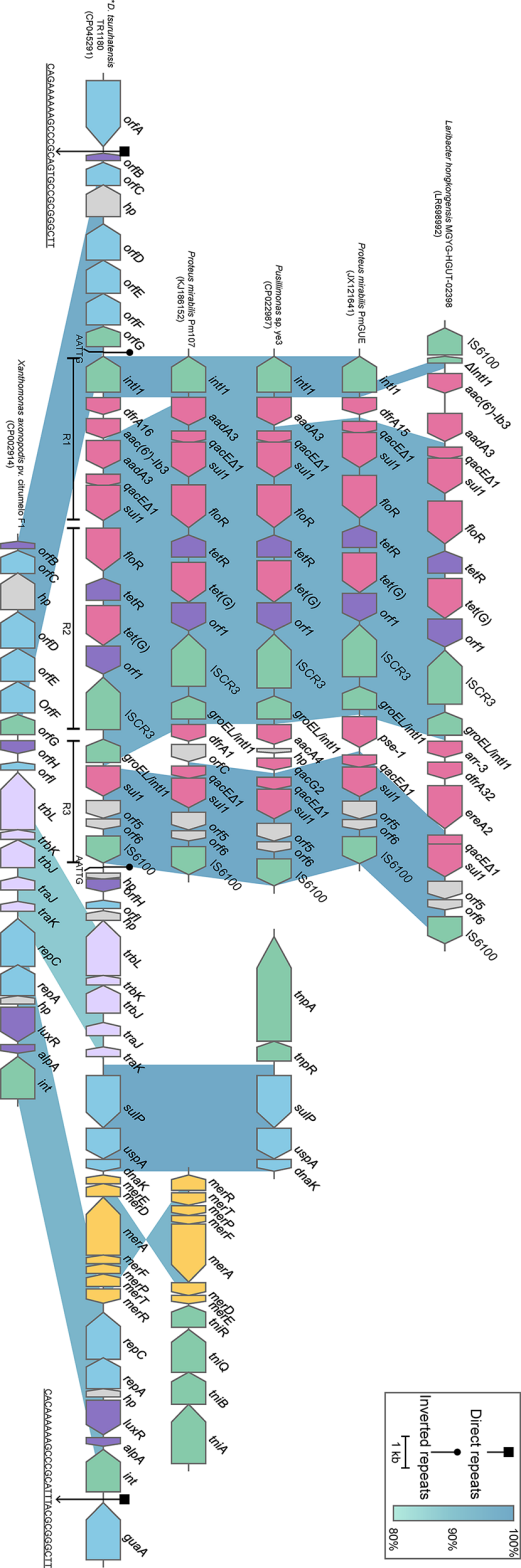
Schematic view of the genomic island integrated in *D. tsuruhatensis* TR1180 and linear comparison with related regions. IRL and IRR sequences flanking the In4-like integron of *D. tsuruhatensis* TR1180 are 5’-TGTCGTTTTCAGAAGACGGCTGCAC-3’, 5’-TGTCATTTTCAGAAGACGACTGCAC-3’ and 5’-GTGCAGTCGTCTTCTGAAAATGACA-3’, respectively. Genes are represented by arrowed boxes and colored based on gene function classification. Uncharacterized genes are illustrated as *orfA* and *orfE*, ATP-binding protein; *orfB* and *orfH*, transcriptional regulator; *orfC*, NADAR family protein; *orfD*, RES family NAD+ phosphorylase; *orfF*, sce7726 family protein; *orfG*, recombinase family protein; and *orfI*, type I toxin-antitoxin system ptaRNA1 family toxin. In4-like elements from *Proteus mirabilis* 09MAS2416, *Salmonella enterica* SC23 and *Salmonella enterica* subsp. *enterica* Z4 are identical to those of *P. mirabilis* Pm107 and hence are not displayed in the figure. *D. tsuruhatensis* TR1180 is indicated by an asterisk.

Comparative genomics analysis of the complete *Delftia* genomes showed that *D. tsuruhatensis* TR1180 harbored a unique 38-kb genomic island containing 49 ORFs (ranging from 4,095 to 4,133 kb in the genome map, **Figures 2** and **4**). The genomic island comprised a phage integrase-encoding gene, replication-associated genes (*repAC*) and partial elements of the conjugal transfer genes (*trbL*-*trbK*-*trbJ*-*traJ*-*traK*), but other essential components (such as the origin of transfer and relaxase gene) for an integrative and conjugative element were not identified (Wozniak and Waldor, 2010). This genomic island was integrated into the chromosome between *orfA* (encoding the ATP-binding protein) and *guaA* (encoding the glutamine-hydrolyzing GMP synthase). A pair of 29-bp direct repeats (DRs) with four nucleotide differences was identified by scanning its upstream and downstream sequences, implying the acquisition of the genomic island was possibly mediated by integrase through site-specific recombination. Further analysis demonstrated that the genomic island consisted of a backbone and several variable regions, including an In4-like integron and two truncated transposons Tn*6050* and Tn*5053*, which conferred resistance to different classes of antibiotics and mercury (**Figure 4** and **Table 3**).

The intact backbone region ranging from *orfB* (encoding the transcriptional regulator) to *orfG* (encoding the recombinase family protein) was found only in the chromosomes of *Xanthomonas axonopodis* pv. citrumelo F1 (with 98.60% nucleotide sequence identity, CP002914), which was reported as a citrus pathogen causing citrus bacterial spot disease (Jalan et al., 2011), and *Ottowia* sp. oral taxon 894 (with 88.44% identity, CP012073). Notably, most of the backbone genes downstream of the In4-like integron of TR1180 could also be identified in *X. axonopodis* pv. citrumelo F1 genome (with 95.47% identity) but not in *Ottowia* sp. genome, which implied that the genome islands of TR1180 and *X. axonopodis* pv. citrumelo F1 might have evolved from a common ancestor (**Figure 4**).

The 14-kb In4-like complex integron within the genomic island lacked the partial IS*6100* element found in In4 and harbored different gene cassettes (In4 carrying an *aacC1*-*orfE*-*aadA2*-*cmlA1* gene cassette array) (Partridge et al., 2001). The whole region was flanked by 25-bp imperfect inverted repeats (noted as IRL and IRR, representing terminal left and right inverted repeats, respectively) and further bracketed by 5-bp DRs (AATTG) at both ends. A BLAST search against the NCBI nucleotide database indicated that the overall structure of the In4-like element in TR1180 resembled that of In4-like elements found in seven bacterial chromosomes (>97% identity and >95% coverage) from three different orders (*Neisseriales*, *Burkholderiales* and *Enterobacterales*) (**Figure 4**). The similarity of In4-like elements among different strains implied that the lateral gene transfer of the In4-like integrons may happen among phylogenetically remote bacteria. Notably, the IS*CR3*-carrying In4-like element was initially described in the *Salmonella* genomic island 1 (SGI1) of *S. enterica* Typhymurium DT104 (Boyd et al., 2000; Boyd et al., 2002). Further analysis showed the In4-like elements of the aforementioned genomes were also located within the SGI1 except for those of TR1180, *Laribacter hongkongensis* MGYG-HGUT-02398 (LR698992) and *Pusillimonas* sp. ye3 (CP022987), which may have been transferred from SGI1. Moreover, alignment of the In4-like integron in TR1180 with that of the other seven sequences showed that the single *dfrA16* was lost in *L. hongkongensis* MGYG-HGUT-02398. In *Proteus mirabilis* PmGUE (JX121641), the *dfrA16*-*aac(6′)-Ib3*-*aadA2* gene cassette array was replaced by *dfrA15* while the deletions of *dfrA16*-*aac(6′)-Ib3* occurred in the other five sequences. To the best of our knowledge, the genomic architecture of a class I integron carrying the *dfrA16-aac(6’)-Ib3*-*aadA2* gene array has not previously been reported. In addition, the class I integron downstream of the IS*CR3* of TR1180 underwent loss of *qacEΔ1* (quaternary ammonium resistance) at the 3’-CS and lacked the variable region in comparison with the retrieved sequences.

Tn*6050* and Tn*5053* were inserted into the genomic island region between the conjugative transfer and replication-related genes with loss of corresponding transposase-encoding genes, which may have occurred during the transposition process. The remnants of Tn*6050* were similar (>99.87% sequence identity) to that of the prototype Tn*6050* located on a megaplasmid from *Cupriavidus metallidurans* CH34 (**Figure 4**), which was isolated from the heavy metal-contaminated sludge in Belgium (Van Houdt et al., 2009). The remnants of Tn*5053* shared a nucleotide sequence identity of 95.15% with the prototype Tn*5053*, as observed in the chromosome of a mercury mine isolate *Xanthomonas* sp. W17 (Kholodii et al., 1993), which was an 8.4-kb transposon containing a *mer* operon (*merRTPFADE*, mercury resistance) (**Figure 4**).

A combination of various MGEs (especially an In4-like integron carrying AMR genes) within the backbone sequence of this genomic island formed a large MDR region with a complex mosaic structure.

## Conclusions

To our knowledge, this study reports novel whole-genomic features and antimicrobial susceptibility patterns of clinical *D. tsuruhatensis* with a fully sequenced genome. Comparative analysis of *Delftia* genomes provided a better understanding of genome divergence among strains of this genus. In addition, all *D. tsuruhatensis* TR1180 AMR genes were embedded in an In4-like integron, which was part of a genomic island, implying that these genes may have been acquired through HGT. Further research should explore the epidemiological characteristics of *D. tsuruhatensis*, which may provide useful information for the clinical control of this bacterium.

## Data Availability Statement

The datasets presented in this study can be found in online repositories. The names of the repository/repositories and accession number(s) can be found in the article/**Supplementary Material**.

## Author Contributions

QB, TX, JL, and JY designed the experiment. CC, WZ, XD, PZ, KZ, DZ, and CQ performed experiments. XL, PL, and KL contributed to analysis the experimental data. CC, JY, and QB wrote the manuscript. KL, QB, and TX critically revised the manuscript. All authors contributed to the article and approved the submitted version.

## Funding

This work was supported by grants from the Natural Science Foundation of Zhejiang Province (LY19C060002 and LY21C010004), the National Natural Science Foundation of China (81960381), and the Science & Technology Project of Inner Mongolia Autonomous Region, China (201802125).

## Conflict of Interest

The authors declare that the research was conducted in the absence of any commercial or financial relationships that could be construed as a potential conflict of interest.
