# Supplementary figures and images for: Genomic Analysis of Delftia tsuruhatensis Strain TR1180 Isolated From A Patient From China With In4-Like Integron-Associated Antimicrobial Resistance
Source: Front Cell Infect Microbiol. 2021 Jun 17;11:663933. doi: 10.3389/fcimb.2021.663933 (PMC8248536; doi:10.3389/fcimb.2021.663933)

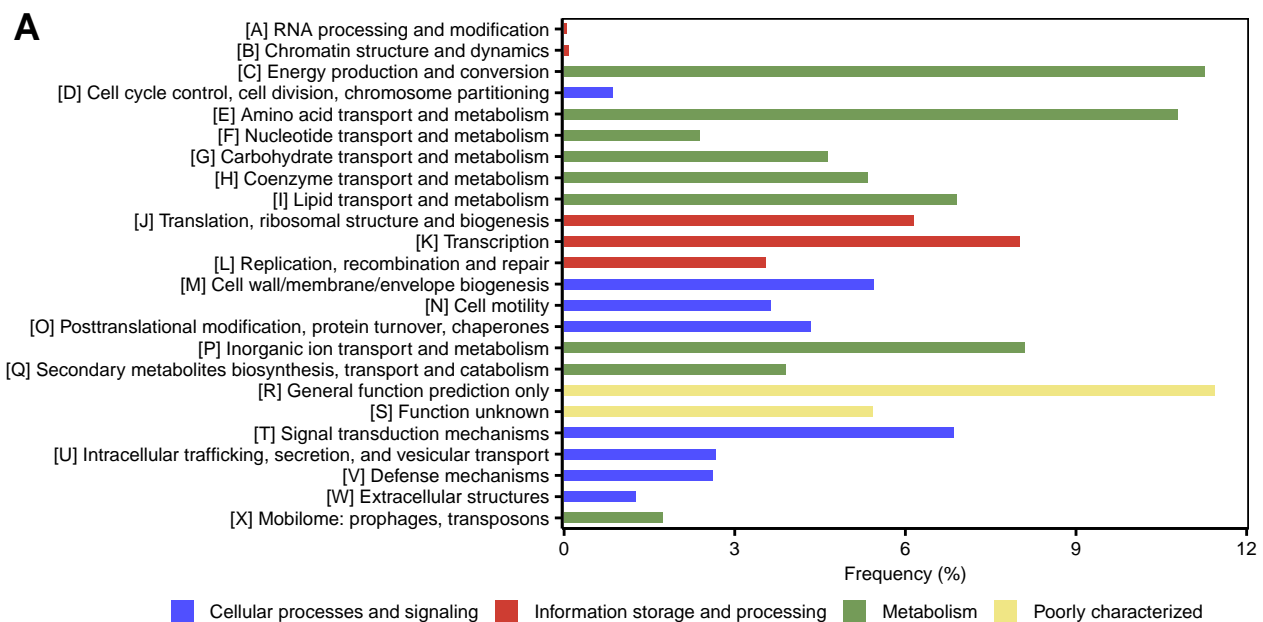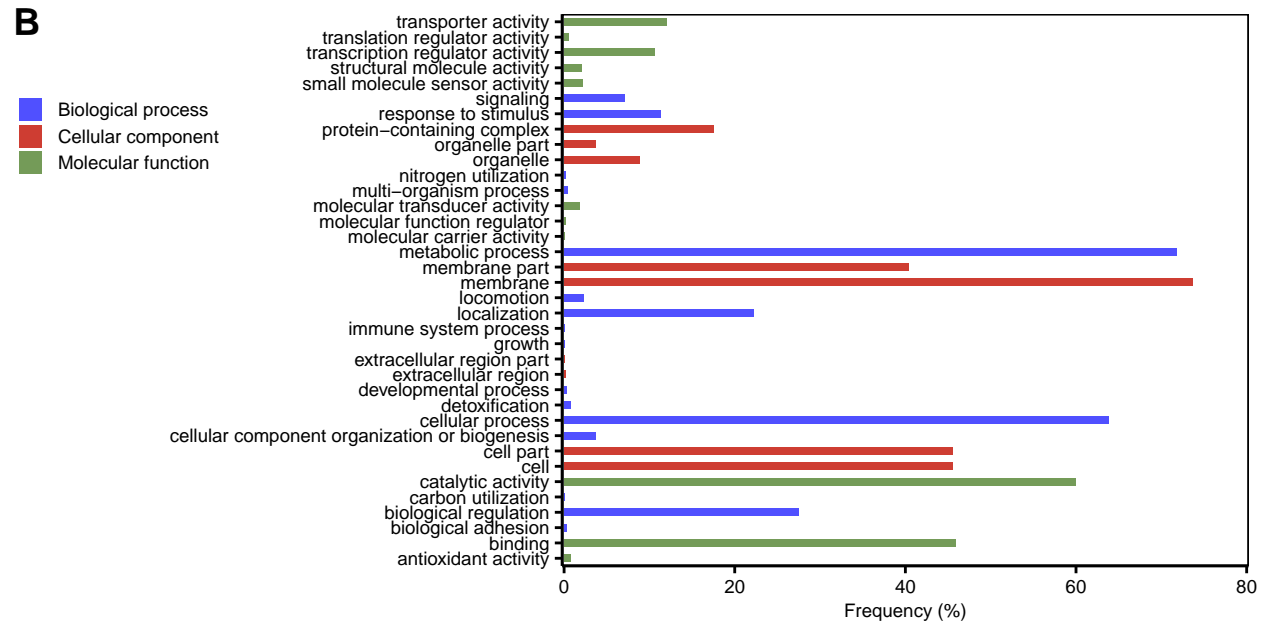

Supplement: Supplementary file 1 [file DataSheet_1.pdf]

**A**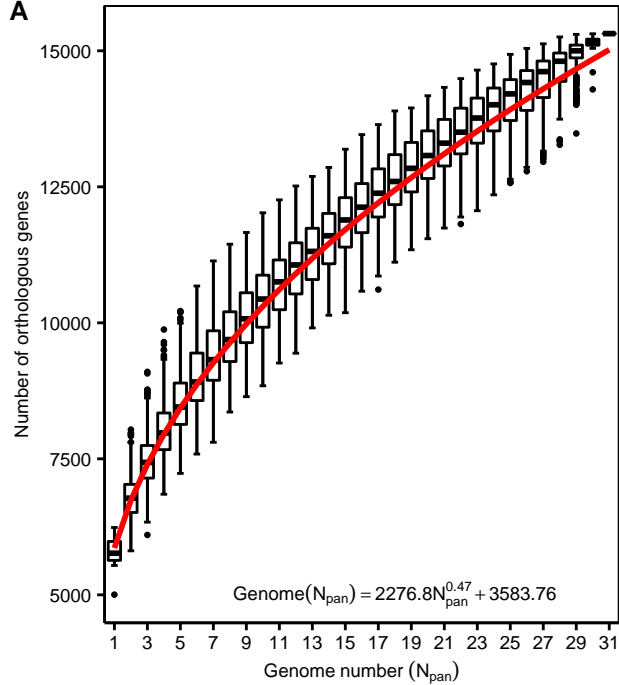**B**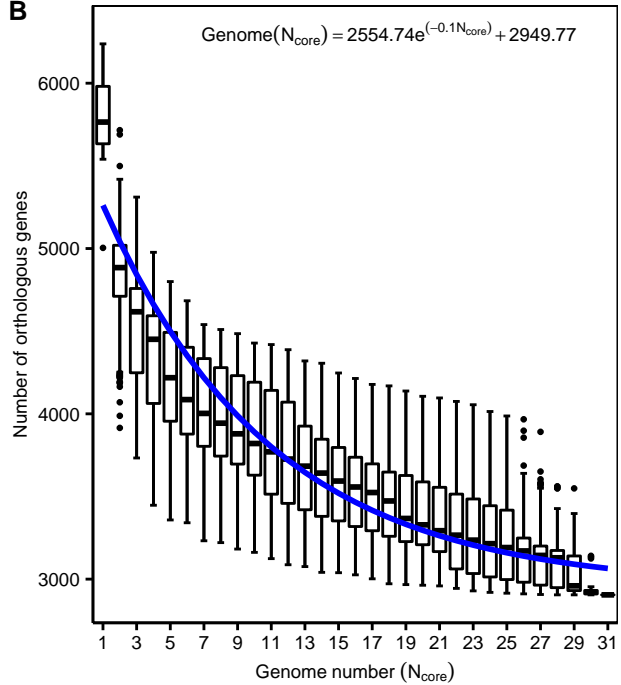

Supplement: Supplementary file 2 [file DataSheet_2.pdf]

unique accessory core

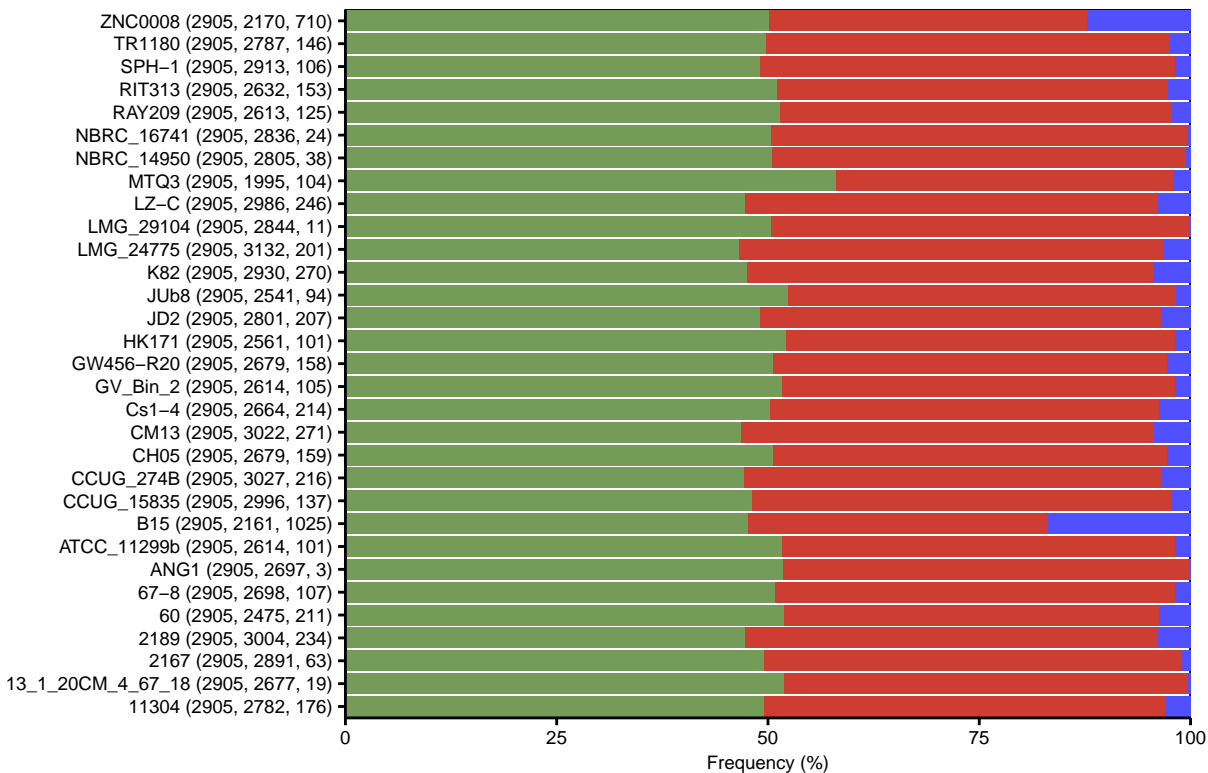

Supplement: Supplementary file 3 [file DataSheet_3.pdf]
