# Supplementary material for: Genomic Analysis of Delftia tsuruhatensis Strain TR1180 Isolated From A Patient From China With In4-Like Integron-Associated Antimicrobial Resistance
Source: Front Cell Infect Microbiol. 2021 Jun 17;11:663933. doi: 10.3389/fcimb.2021.663933 (PMC8248536; doi:10.3389/fcimb.2021.663933)
Supplement: Supplementary file 4 [file DataSheet_4.docx]

**Figure S1** Functional classification of gene products encoded by *D. tsuruhatensis* TR1180 based on the (A) COG database and (B) GO analysis.

**Figure S2** Accumulation curves for the (A) pan-genome and (B) core genome of *Delftia*, respectively. The boxes denote the number of orthologous clusters discovered with the sequential addition of new genomes. The curve is a fitted-line model generated by PanGP, representing the relationship between the genome number and orthologous cluster number.

**Figure S3** Percentage of core, dispensable, and unique genes in each of the 31 *Delftia* genomes, with the corresponding number of them sequentially put in parentheses.
