# Supplementary material for: Genomic Analysis of Delftia tsuruhatensis Strain TR1180 Isolated From A Patient From China With In4-Like Integron-Associated Antimicrobial Resistance
Source: Front Cell Infect Microbiol. 2021 Jun 17;11:663933. doi: 10.3389/fcimb.2021.663933 (PMC8248536; doi:10.3389/fcimb.2021.663933)
Supplement: Supplementary file 5 [file Table_1.docx]

**Table S1** The information of 31 *Delftia* strains used in this study

| Organism | Strain | Size (Mb) | Assembly Accession | WGS | Release Date | Pubmed ID |
| --- | --- | --- | --- | --- | --- | --- |
| *D. tsuruhatensis* | MTQ3 | 5.74 | GCA_001021565.1 | LCZH01 | 2015/6/8 | 26251486 |
| *D. tsuruhatensis* | NBRC 16741 | 6.61 | GCA_001571325.1 | BCTO01 | 2016/2/18 | - |
| *D. tsuruhatensis* | CM13 | 7.20 | GCA_001753225.1 | - | 2016/10/6 | - |
| *D. tsuruhatensis* | 11304 | 6.62 | GCA_004353655.1 | SMMJ01 | 2019/3/16 | - |
| *D. tsuruhatensis* | TR1180 | 6.71 | GCA_009362995.1 | - | 2019/10/27 | - |
| *D. tsuruhatensis* | LMG 29104 | 6.64 | GCA_900111975.1 | FOKN01 | 2016/10/31 | - |
| *D. lacustris* | LZ-C | 6.98 | GCA_001017795.1 | JNCQ01 | 2015/6/2 | 26589947 |
| *D. lacustris* | LMG 24775 | 7.17 | GCA_900107225.1 | FNPE01 | 2016/10/22 | - |
| *D. acidovorans* | SPH-1 | 6.77 | GCA_000018665.1 | - | 2007/11/16 | 15240283 |
| *D. acidovorans* | CCUG 274B | 6.95 | GCA_000411195.1 | AGYX01 | 2013/6/13 | - |
| *D. acidovorans* | CCUG 15835 | 6.82 | GCA_000411215.1 | AGYY01 | 2013/6/14 | - |
| *D. acidovorans* | 2167 | 6.78 | GCA_000741825.1 | JOUB01 | 2014/8/18 | 25237026 |
| *D. acidovorans* | NBRC 14950 | 6.63 | GCA_001598795.1 | BCZP01 | 2016/3/11 | - |
| *D. acidovorans* | ANG1 | 6.56 | GCA_001969245.1 | - | 2017/1/23 | - |
| *D. acidovorans* | RAY209 | 6.53 | GCA_002443055.1 | - | 2017/10/4 | - |
| *D. acidovorans* | B15 | 6.55 | GCA_002751115.2 | PEKB02 | 2017/11/5 | - |
| *D. acidovorans* | JUb8 | 6.35 | GCA_003752215.1 | RJUY01 | 2018/11/15 | - |
| *D. acidovorans* | 2189 | 6.97 | GCA_900078185.2 | FLCF02 | 2018/3/22 | - |
| *D. acidovorans* | ATCC 11299b | 6.44 | GCA_900215545.1 | OCRO01 | 2017/9/19 | - |
| *D.* sp. | Cs1-4 | 6.69 | GCA_000214395.1 | - | 2011/5/23 | - |
| *D.* sp. | RIT313 | 6.70 | GCA_000632165.1 | JFYT01 | 2014/4/14 | 24812212 |
| *D.* sp. | ZNC0008 | 6.33 | GCA_000799035.1 | JTKZ01 | 2014/12/8 | - |
| *D.* sp. | GW456-R20 | 6.66 | GCA_001622915.1 | LWCN01 | 2016/4/18 | - |
| *D.* sp. | JD2 | 6.76 | GCA_001682645.1 | LFJT01 | 2016/7/8 | - |
| *D.* sp. | HK171 | 6.43 | GCA_001886655.1 | - | 2016/11/28 | - |
| *D.* sp. | 67-8 | 6.48 | GCA_001898745.1 | MKUX01 | 2016/12/9 | - |
| *D.* sp. | 13_1_20CM_4_67_18 | 6.41 | GCA_001919935.1 | MNIU01 | 2016/12/23 | 27843720 |
| *D.* sp. | K82 | 7.11 | GCA_002193035.1 | MYFL01 | 2017/6/14 | - |
| *D.* sp. | 60 | 6.38 | GCA_002754395.1 | PEFD01 | 2017/11/3 | - |
| *D.* sp. | GV_Bin_2 | 6.45 | GCA_009360865.1 | SPEK01 | 2019/10/25 | - |
| *D.* sp. | CH05 | 6.63 | GCA_009827015.1 | WUMG01 | 2020/1/1 | - |
