# Supplementary material for: Genomic Analysis of Delftia tsuruhatensis Strain TR1180 Isolated From A Patient From China With In4-Like Integron-Associated Antimicrobial Resistance
Source: Front Cell Infect Microbiol. 2021 Jun 17;11:663933. doi: 10.3389/fcimb.2021.663933 (PMC8248536; doi:10.3389/fcimb.2021.663933)
Supplement: Supplementary file 6 [file Table_2.docx]

**Table S2** Pathway enrichment analysis of the core genes

| Pathway id | Pathway description | In core genome | Core genes | In pan-genome | Pan genes | Pvalue | FDR |
| --- | --- | --- | --- | --- | --- | --- | --- |
| ko03010 | Ribosome | 51 | 1000 | 55 | 1912 | 5.02E-11 | 4.92E-09 |
| ko00190 | Oxidative phosphorylation | 45 | 1000 | 56 | 1912 | 9.59E-06 | 0.000469829 |
| ko00230 | Purine metabolism | 52 | 1000 | 77 | 1912 | 0.004134324 | 0.126685785 |
| ko00970 | Aminoacyl-tRNA biosynthesis | 23 | 1000 | 30 | 1912 | 0.005170848 | 0.126685785 |
| ko03060 | Protein export | 14 | 1000 | 17 | 1912 | 0.010308906 | 0.202054559 |
| ko00730 | Thiamine metabolism | 11 | 1000 | 13 | 1912 | 0.016741229 | 0.272895967 |
| ko02025 | Biofilm formation - Pseudomonas aeruginosa | 32 | 1000 | 47 | 1912 | 0.019492569 | 0.272895967 |
| ko05111 | Biofilm formation - Vibrio cholerae | 19 | 1000 | 26 | 1912 | 0.024756376 | 0.300840204 |
| ko00740 | Riboflavin metabolism | 10 | 1000 | 12 | 1912 | 0.027628182 | 0.300840204 |
| ko00010 | Glycolysis / Gluconeogenesis | 23 | 1000 | 33 | 1912 | 0.031430916 | 0.308022975 |
| ko01501 | beta-Lactam resistance | 15 | 1000 | 21 | 1912 | 0.059590361 | 0.525461144 |
| ko00720 | Carbon fixation pathways in prokaryotes | 22 | 1000 | 33 | 1912 | 0.066984339 | 0.525461144 |
| ko00760 | Nicotinate and nicotinamide metabolism | 19 | 1000 | 28 | 1912 | 0.069704029 | 0.525461144 |
| ko00300 | Lysine biosynthesis | 14 | 1000 | 20 | 1912 | 0.084435944 | 0.591051609 |
| ko00290 | Valine, leucine and isoleucine biosynthesis | 15 | 1000 | 22 | 1912 | 0.098455107 | 0.643240034 |
| ko03070 | Bacterial secretion system | 25 | 1000 | 40 | 1912 | 0.125779222 | 0.742667495 |
| ko00710 | Carbon fixation in photosynthetic organisms | 11 | 1000 | 16 | 1912 | 0.14176104 | 0.742667495 |
| ko00195 | Photosynthesis | 8 | 1000 | 11 | 1912 | 0.145021654 | 0.742667495 |
| ko00380 | Tryptophan metabolism | 19 | 1000 | 30 | 1912 | 0.150232286 | 0.742667495 |
| ko00260 | Glycine, serine and threonine metabolism | 29 | 1000 | 48 | 1912 | 0.16019396 | 0.742667495 |
| ko04122 | Sulfur relay system | 9 | 1000 | 13 | 1912 | 0.172100937 | 0.742667495 |
| ko00362 | Benzoate degradation | 17 | 1000 | 27 | 1912 | 0.178252138 | 0.742667495 |
| ko03410 | Base excision repair | 10 | 1000 | 15 | 1912 | 0.195996865 | 0.742667495 |
| ko04112 | Cell cycle - Caulobacter | 10 | 1000 | 15 | 1912 | 0.195996865 | 0.742667495 |
| ko00790 | Folate biosynthesis | 19 | 1000 | 31 | 1912 | 0.204021327 | 0.742667495 |
| ko05016 | Huntington disease | 7 | 1000 | 10 | 1912 | 0.21159627 | 0.742667495 |
| ko00860 | Porphyrin and chlorophyll metabolism | 25 | 1000 | 42 | 1912 | 0.214786046 | 0.742667495 |
| ko00130 | Ubiquinone and other terpenoid-quinone biosynthesis | 11 | 1000 | 17 | 1912 | 0.217279277 | 0.742667495 |
| ko00020 | Citrate cycle (TCA cycle) | 16 | 1000 | 26 | 1912 | 0.226828546 | 0.742667495 |
| ko00400 | Phenylalanine, tyrosine and tryptophan biosynthesis | 21 | 1000 | 35 | 1912 | 0.227347192 | 0.742667495 |
| ko00900 | Terpenoid backbone biosynthesis | 12 | 1000 | 19 | 1912 | 0.236403657 | 0.747340594 |
| ko00910 | Nitrogen metabolism | 15 | 1000 | 25 | 1912 | 0.28406563 | 0.869950992 |
| ko00360 | Phenylalanine metabolism | 26 | 1000 | 46 | 1912 | 0.334221916 | 0.986686147 |
| ko00627 | Aminobenzoate degradation | 8 | 1000 | 13 | 1912 | 0.350341011 | 0.986686147 |
| ko00250 | Alanine, aspartate and glutamate metabolism | 21 | 1000 | 37 | 1912 | 0.35238791 | 0.986686147 |
| ko00310 | Lysine degradation | 12 | 1000 | 21 | 1912 | 0.41180054 | 0.998189692 |
| ko00333 | Prodigiosin biosynthesis | 6 | 1000 | 10 | 1912 | 0.434527468 | 0.998189692 |
| ko02026 | Biofilm formation - Escherichia coli | 15 | 1000 | 27 | 1912 | 0.44290502 | 0.998189692 |
| ko02040 | Flagellar assembly | 25 | 1000 | 46 | 1912 | 0.448478752 | 0.998189692 |
| ko00643 | Styrene degradation | 7 | 1000 | 12 | 1912 | 0.450527701 | 0.998189692 |
| ko03420 | Nucleotide excision repair | 7 | 1000 | 12 | 1912 | 0.450527701 | 0.998189692 |
| ko00030 | Pentose phosphate pathway | 14 | 1000 | 26 | 1912 | 0.516647352 | 0.998189692 |
| ko00220 | Arginine biosynthesis | 14 | 1000 | 26 | 1912 | 0.516647352 | 0.998189692 |
| ko04146 | Peroxisome | 15 | 1000 | 28 | 1912 | 0.523017991 | 0.998189692 |
| ko00650 | Butanoate metabolism | 29 | 1000 | 55 | 1912 | 0.529758012 | 0.998189692 |
| ko00240 | Pyrimidine metabolism | 22 | 1000 | 42 | 1912 | 0.558718812 | 0.998189692 |
| ko00460 | Cyanoamino acid metabolism | 6 | 1000 | 11 | 1912 | 0.562238211 | 0.998189692 |
| ko00562 | Inositol phosphate metabolism | 6 | 1000 | 11 | 1912 | 0.562238211 | 0.998189692 |
| ko00072 | Synthesis and degradation of ketone bodies | 7 | 1000 | 13 | 1912 | 0.567412172 | 0.998189692 |
| ko00561 | Glycerolipid metabolism | 7 | 1000 | 13 | 1912 | 0.567412172 | 0.998189692 |
| ko00280 | Valine, leucine and isoleucine degradation | 25 | 1000 | 48 | 1912 | 0.57093205 | 0.998189692 |
| ko00564 | Glycerophospholipid metabolism | 11 | 1000 | 21 | 1912 | 0.584991397 | 0.998189692 |
| ko00620 | Pyruvate metabolism | 29 | 1000 | 56 | 1912 | 0.585414578 | 0.998189692 |
| ko00340 | Histidine metabolism | 13 | 1000 | 25 | 1912 | 0.592521896 | 0.998189692 |
| ko00550 | Peptidoglycan biosynthesis | 13 | 1000 | 25 | 1912 | 0.592521896 | 0.998189692 |
| ko03440 | Homologous recombination | 12 | 1000 | 24 | 1912 | 0.667877298 | 0.998189692 |
| ko03030 | DNA replication | 9 | 1000 | 18 | 1912 | 0.6681115 | 0.998189692 |
| ko00350 | Tyrosine metabolism | 15 | 1000 | 30 | 1912 | 0.66996674 | 0.998189692 |
| ko00071 | Fatty acid degradation | 17 | 1000 | 34 | 1912 | 0.672023122 | 0.998189692 |
| ko05132 | Salmonella infection | 6 | 1000 | 12 | 1912 | 0.673948722 | 0.998189692 |
| ko00960 | Tropane, piperidine and pyridine alkaloid biosynthesis | 5 | 1000 | 10 | 1912 | 0.678653934 | 0.998189692 |
| ko00480 | Glutathione metabolism | 18 | 1000 | 37 | 1912 | 0.731072351 | 0.998189692 |
| ko00640 | Propanoate metabolism | 24 | 1000 | 49 | 1912 | 0.731437329 | 0.998189692 |
| ko00410 | beta-Alanine metabolism | 9 | 1000 | 19 | 1912 | 0.746548353 | 0.998189692 |
| ko00450 | Selenocompound metabolism | 7 | 1000 | 15 | 1912 | 0.75739167 | 0.998189692 |
| ko00660 | C5-Branched dibasic acid metabolism | 7 | 1000 | 15 | 1912 | 0.75739167 | 0.998189692 |
| ko04714 | Thermogenesis | 6 | 1000 | 13 | 1912 | 0.765265765 | 0.998189692 |
| ko00630 | Glyoxylate and dicarboxylate metabolism | 33 | 1000 | 68 | 1912 | 0.775803394 | 0.998189692 |
| ko00770 | Pantothenate and CoA biosynthesis | 16 | 1000 | 34 | 1912 | 0.785520073 | 0.998189692 |
| ko02010 | ABC transporters | 106 | 1000 | 213 | 1912 | 0.804835115 | 0.998189692 |
| ko03018 | RNA degradation | 9 | 1000 | 20 | 1912 | 0.81110441 | 0.998189692 |
| ko00051 | Fructose and mannose metabolism | 8 | 1000 | 18 | 1912 | 0.817868959 | 0.998189692 |
| ko01503 | Cationic antimicrobial peptide (CAMP) resistance | 9 | 1000 | 21 | 1912 | 0.862342085 | 0.998189692 |
| ko04621 | NOD-like receptor signaling pathway | 4 | 1000 | 10 | 1912 | 0.863913894 | 0.998189692 |
| ko00983 | Drug metabolism - other enzymes | 8 | 1000 | 19 | 1912 | 0.869738491 | 0.998189692 |
| ko00670 | One carbon pool by folate | 7 | 1000 | 17 | 1912 | 0.878328276 | 0.998189692 |
| ko00540 | Lipopolysaccharide biosynthesis | 10 | 1000 | 24 | 1912 | 0.895408038 | 0.998189692 |
| ko00680 | Methane metabolism | 17 | 1000 | 39 | 1912 | 0.896618599 | 0.998189692 |
| ko05418 | Fluid shear stress and atherosclerosis | 8 | 1000 | 20 | 1912 | 0.908827878 | 0.998189692 |
| ko00270 | Cysteine and methionine metabolism | 23 | 1000 | 53 | 1912 | 0.927291858 | 0.998189692 |
| ko00061 | Fatty acid biosynthesis | 15 | 1000 | 36 | 1912 | 0.927705845 | 0.998189692 |
| ko00780 | Biotin metabolism | 9 | 1000 | 23 | 1912 | 0.931179655 | 0.998189692 |
| ko03430 | Mismatch repair | 9 | 1000 | 23 | 1912 | 0.931179655 | 0.998189692 |
| ko04212 | Longevity regulating pathway - worm | 8 | 1000 | 21 | 1912 | 0.93743452 | 0.998189692 |
| ko03320 | PPAR signaling pathway | 7 | 1000 | 19 | 1912 | 0.944256325 | 0.998189692 |
| ko00920 | Sulfur metabolism | 18 | 1000 | 44 | 1912 | 0.953984276 | 0.998189692 |
| ko01524 | Platinum drug resistance | 5 | 1000 | 15 | 1912 | 0.9597033 | 0.998189692 |
| ko05204 | Chemical carcinogenesis | 5 | 1000 | 15 | 1912 | 0.9597033 | 0.998189692 |
| ko05200 | Pathways in cancer | 6 | 1000 | 18 | 1912 | 0.969114017 | 0.998189692 |
| ko00330 | Arginine and proline metabolism | 15 | 1000 | 39 | 1912 | 0.972212762 | 0.998189692 |
| ko02024 | Quorum sensing | 54 | 1000 | 122 | 1912 | 0.973241782 | 0.998189692 |
| ko02030 | Bacterial chemotaxis | 17 | 1000 | 44 | 1912 | 0.976855272 | 0.998189692 |
| ko02020 | Two-component system | 95 | 1000 | 209 | 1912 | 0.985090244 | 0.998189692 |
| ko04626 | Plant-pathogen interaction | 3 | 1000 | 12 | 1912 | 0.987255901 | 0.998189692 |
| ko05225 | Hepatocellular carcinoma | 3 | 1000 | 13 | 1912 | 0.992945711 | 0.998189692 |
| ko00980 | Metabolism of xenobiotics by cytochrome P450 | 4 | 1000 | 16 | 1912 | 0.993712438 | 0.998189692 |
| ko00982 | Drug metabolism - cytochrome P450 | 4 | 1000 | 16 | 1912 | 0.993712438 | 0.998189692 |
| ko00520 | Amino sugar and nucleotide sugar metabolism | 12 | 1000 | 39 | 1912 | 0.998189692 | 0.998189692 |

^*^Column 3 denotes the number of core genes involved in each pathway; Column 4 denotes the number of core genes involved in all metabolic pathways; Column 5 denotes the number of pan genes involved in each pathway; Column 6 denotes the number of pan genes involved in all metabolic pathways.
