# Supplementary material for: Genomic Analysis of Delftia tsuruhatensis Strain TR1180 Isolated From A Patient From China With In4-Like Integron-Associated Antimicrobial Resistance
Source: Front Cell Infect Microbiol. 2021 Jun 17;11:663933. doi: 10.3389/fcimb.2021.663933 (PMC8248536; doi:10.3389/fcimb.2021.663933)
Supplement: Supplementary file 7 [file Table_3.docx]

**Table S3** Enrichment analysis of virulence and TCS in the core genome of *Delftia* strains

| Gene classification | In core genome | In pan-genome | Core genes | Pan genes | Pvalue |
| --- | --- | --- | --- | --- | --- |
| Virulence gene | 20 | 33 | 2905 | 15316 | 1.52E-07 |
| TCS gene | 72 | 157 | 2905 | 15316 | 1.14E-14 |

^*^Pvalue reflects the degree of enrichment of virulence and TCS genes in the core genome compared to these in the pan genome, which was performed by Fisher′s exact test.
